# Stumbling reactions in hypo and hyper gravity – muscle synergies are robust across different perturbations of human stance during parabolic flights

**DOI:** 10.1038/s41598-019-47091-x

**Published:** 2019-07-19

**Authors:** Janek Holubarsch, Michael Helm, Steffen Ringhof, Albert Gollhofer, Kathrin Freyler, Ramona Ritzmann

**Affiliations:** 1 Department of Sport and Sport Science, University of Freiburg, Freiburg, Germany; 2 Praxisklinik Rennbahn AG, Muttenz, Switzerland

**Keywords:** Motor control, Computational science

## Abstract

The control of bipedal stance and the capacity to regain postural equilibrium after its deterioration in variable gravities are crucial prerequisites for manned space missions. With an emphasize on natural orthograde posture, computational techniques synthesize muscle activation patterns of high complexity to a simple synergy organization. We used nonnegative matrix factorization to identify muscle synergies during postural recovery responses in human and to examine the functional significance of such synergies for hyper-gravity (1.75 g) and hypo-gravity (0.25 g). Electromyographic data were recorded from leg, trunk and arm muscles of five human exposed to five modes of anterior and posterior support surface translations during parabolic flights including transitional g-levels of 0.25, 1 and 1.75 g. Results showed that in 1 g four synergies accounted for 99% of the automatic postural response across all muscles and perturbation directions. Each synergy in 1 g was correlated to the corresponding one in 0.25 and 1.75 g. This study therefore emphasizes the similarity of the synergy organization of postural recovery responses in Earth, hypo- and hyper-gravity conditions, indicating that the muscle synergies and segmental strategies acquired under terrestrial habits are robust and persistent across variable and acute changes in gravity levels.

## Introduction

The gravitational force on Earth has remained constant since the formation of the planet^1^. Living species have evolved to master and rely upon gravity equal to 1 g and adapted motor control to the physical environmental characteristic for our home planet^2^. Gravity determines our daily movement including upright stance and bipedal locomotion^3^. But what happens if the gravitational force is changed below or beyond 1 g?

Particularly in space science, the adaptation of motor control to spontaneous changes in gravity is of major significance^4^. However, there is only minor scientific evidence with reference to the capacity of the central nervous system (CNS) to accurately anticipate the gravity level^5^ and to coordinate muscle onset and activation between the two legs^6^. A few studies have been executed in paradigms of posture control^3^. With an emphasize on distal muscle groups, authors demonstrated gradually increased muscle activities, facilitation of spinal reflexes and delayed onset latencies with progressively increasing gravity during upright stance^7^. Some of these findings are quite intuitive, since compensating for gravitational forces and shifts of the center of mass (COM) presupposes an adequate muscle’s level of activation, which depends on the loading force and thus is proportional to gravitation^6^. However, the regulation of human posture and locomotion requires not only sufficient muscular activity, but also an accurate coordination of the muscle groups involved. Particularly slips or stumbles call for a precise neuronal control of the skeletal muscles in temporal and spatial domains, transmitting the force to the skeleton in order to regain postural equilibrium after its deterioration^8^.

Considering the complexity of the postural control system, including diverse strategies and mechanisms along with a multitude of postural muscles available, there would be a plethora of ways to activate and coordinate the muscle groups^9^. By using computational techniques, it has been assumed that the activation patterns of postural muscles can be synthesized into synergy organizations in both animal^10^ and human models^11^. These muscle synergies have been proposed as a way for the CNS to simplify this complex process of generating motor commands^12^. Accordingly, the CNS is suggested to not control each muscle independently, but to combine certain muscles into functional units, which are inhibited or facilitated simultaneously or successively as a whole group^13,14^. Since recruiting changes between agonistically and antagonistically acting muscles are possible, only a small set of control variables could be advantageous for the control and regulation of body oscillations^10^.

The nonnegative matrix factorization (NNMF) approach has been validated in identifying muscle synergies during postural responses to examine the functional significance of such synergies for natural recovery responses^9,11^. These results suggest that neither a simple reflex mechanism nor a fixed synergy organization is adequate to explain the muscle activation patterns observed in postural control tasks^8,15^. Instead, a flexible continuum of muscle synergies that are modifiable in a time- and task-dependent manner is suggested to be used for equilibrium control^16^. However, this synergy organization seems to be subject-specific^17^, and there is increasing evidence for the robustness of those synergies across a variety of biomechanical contexts^17,18^, indicating common neural mechanisms for reactive balance across different tasks^9^.

However, whereas the synergy organization of postural muscles is well established for balance control during quiet stance and walking as well as for recovery responses after multi-directional platform perturbations, yet there is no evidence as to whether postural muscle synergies are consistent across different gravitational conditions. Therefore, the purpose of this experiment was to examine the muscle synergy organization of postural recovery responses in Earth, hypo- and in hyper-gravity conditions and by this means to test whether the synergies were related to the biomechanical variable of the torque between the limb and the support surface.

To do so, a single-group repeated-measures design including five participants was used to examine the muscle synergy organization of postural recovery responses in Earth (1 g), hypo- (0.25 g) and hyper-gravity (1.75 g) induced by parabolic flights. Electromyographic (EMG) data were recorded while subjects stood barefoot on a two-belt treadmill which randomly generated either bilateral or unilateral left or split perturbations. Prior to perturbations, subjects maintained an upright orthograde posture, arms hanging at the lateral sides and weight equally distributed over both feet. Muscles synergies were extracted using NNMF and were compared between g-levels using correlation coefficients.

Based on pervious findings confirming the robustness of muscle synergies^17^, we hypothesized that gravity has only a minor impact on the muscle synergies used to regain postural equilibrium after its perturbation. We expected the muscle synergies and segmental strategy to be consistent across the gravity levels ranging from 0.25 g to 1 g to 1.75 g and perturbations of human stance.

## Results

### Physics of treadmill perturbation

The treadmill displacement, its maximum speed and acceleration as well as the impulse duration for the bilateral, unilateral and split perturbations are displayed in Table 1. The physics were statistically equal between the three perturbation conditions over the three gravity levels.

**Table 1:** Physical parameters of treadmill pulses.

	Bilateral posterior		Bilateral anterior		Unilateral posterior		Unilateral anterior		Split	
	Left	Right	Left	Right	Left	Right	Left	Right	Left	Right
d (cm)	−10 ± 0^≈^	−10 ± 0^≈^	−10 ± 0^≈^	—	−10 ± 0^≈^	−10 ± 0^≈^	−10 ± 0^≈^	—	−10 ± 0^≈^	−10 ± 0^≈^
t (ms)	214 ± 5^≈^	210 ± 2^≈^	214 ± 4^≈^	—	212 ± 3^≈^	211 ± 4^≈^	209 ± 5^≈^	—	211 ± 2^≈^	210 ± 4^≈^
v_max_ (m/s)	0.8 ± 0.0^≈^	0.8 ± 0.0^≈^	0.8 ± 0.0^≈^	—	0.8 ± 0.0^≈^	0.8 ± 0.0^≈^	0.8 ± 0.0^≈^	—	0.8 ± 0.0^≈^	0.8 ± 0.0^≈^
a_max_ (m/s^2^)	22 ± 3^≈^	21 ± 1^≈^	22 ± 3^≈^	—	21 ± 1^≈^	22 ± 0^≈^	21 ± 1^≈^	—	22 ± 3^≈^	23 ± 1

Values are absolute values. (d = distance [cm]; t = impulse duration [ms]; v_max_ = maximum speed [m/s]; a_max_ = maximal acceleration of the treadmill [m/s²]).

### Synergies

The least number of synergies that fulfilled the VAF criteria was 5 (VAF_tot_ = 0.9972, minimum of all VAF_cond_ = 0.9046). In contrast, the factorization consisting of 4 synergies provided a matchable reconstruction quality with VAF_tot_ = 0.9929 and VAF_cond_ > 0.9 except for two (of 25) conditions lower than the critical value (0.6865 and 0.7026). That is why an additional criterion was applied: The corresponding synergy vectors of repeated algorithms of the same data and number of synergies were compared by Pearson’s correlation coefficient as following:

For s = 5 synergies, the reproduction calculated 4 similar (r_Wi_ > r_crit_ = 0.798, N = 9, p = 0.01) and one altering vector (r_W5_ < r_crit_ = 0.798), whereas for s = 4 synergy vectors were reproduced quite identical (Table 2). For that reason, we decided to use 4 synergies. High correlation coefficients are indicative for algorithm consistency.

**Table 2:** Correlation coefficients of repeated calculations.

4 synergies	5 synergies
r_W1_ = 0.990	r_W1_ = 0.923
r_W2_ = 0.998	r_W2_ = 0.939
r_W3_ = 0.985	r_W3_ = 0.817
r_W4_ = 0.981	r_W4_ = 0.808
	r_W5_ = 0.270

Correlation coefficients between the corresponding synergy vectors W_i_ of two sets of synergies extracted both from the original data using 4 synergies (first column) and 5 synergies (right column).

**Figure 1:**
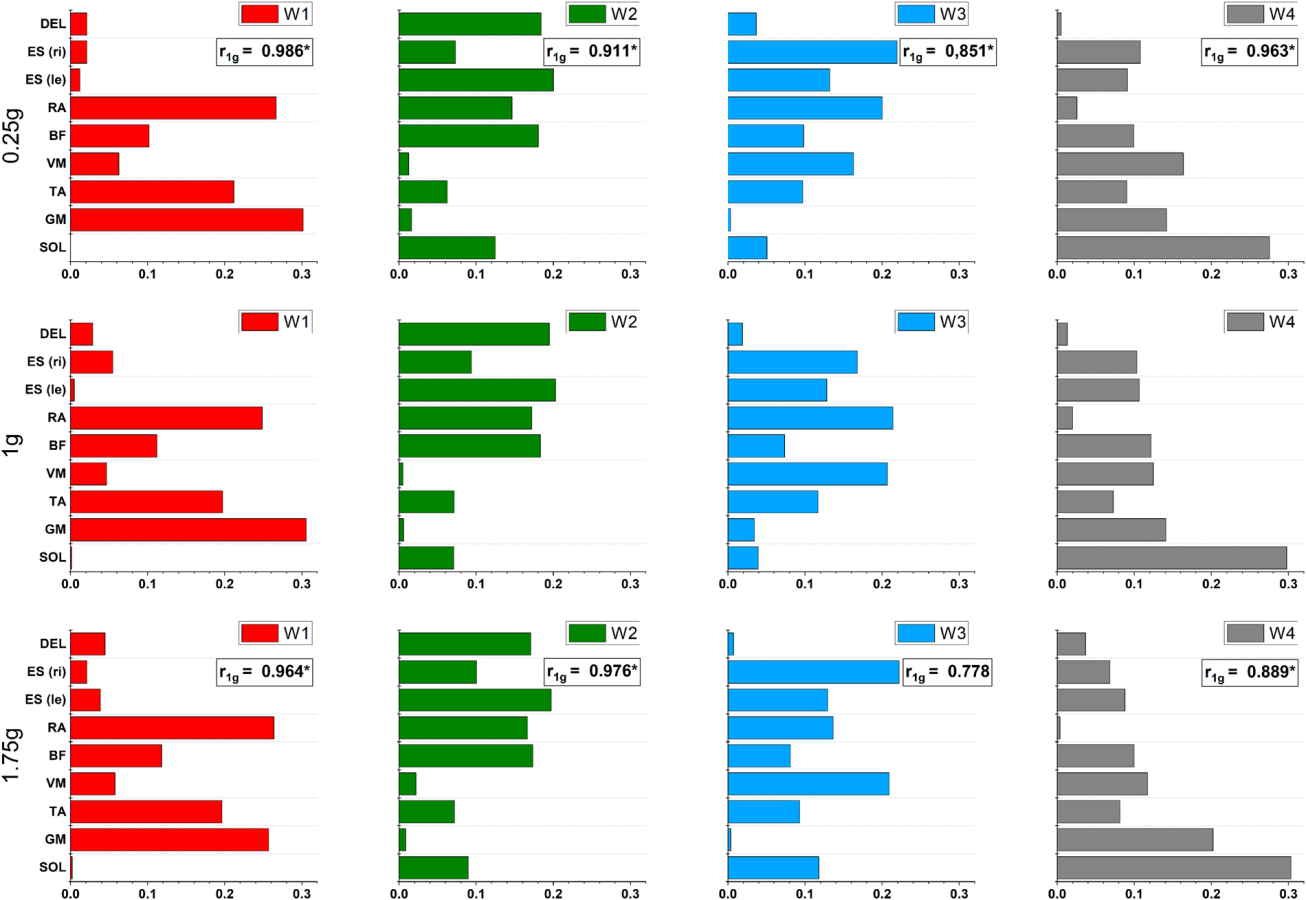
Four muscle synergies extracted for 5 subjects, perturbations and reflex phases in 1 g (top), 0.25 g (middle) and 1.75 g (bottom). Values on the x-axis refer to the relative muscle contribution to each synergy, not to maximum voluntary contraction. Correlation coefficients r_1g_ (boxes) indicate the correlation of the synergy vectors in 0.25 and 1.75 g to the corresponding synergy vector in 1 g (same color). Coefficients above critical value (r_crit_ = 0.798, signed with *) are considered as similar. SOL: m. soleus, GM: m. gastrocnemius medialis, TA: m. tibialis anterior, VM: m. vastus medialis, BF: m. biceps femoris, RA: m. rectus abdominis, ES (le): m. erector spinae left, ES (ri): m. erector spinae right, DEL: m. deltoideus.

Figure 1 shows the 4 synergies in 1 g and their corresponding synergies from 0.25 g and 1.75 g across the five perturbations. The synergies extracted from 0.25 g and 1.75 g data are constituted that each synergy of one condition corresponds with only one of the 1 g condition. Correlation coefficients indicate a significant similarity between all corresponding synergies of the 0.25 g and 1 g condition and between 3 corresponding synergies of the 1.75 g and 1 g condition (r_1g_ > 0.798, N = 9, p = 0.01). The non-significant pair of synergies (W3) reaches a relatively high correlation as well (r_1g_ = 0.778).

**Figure 2:**
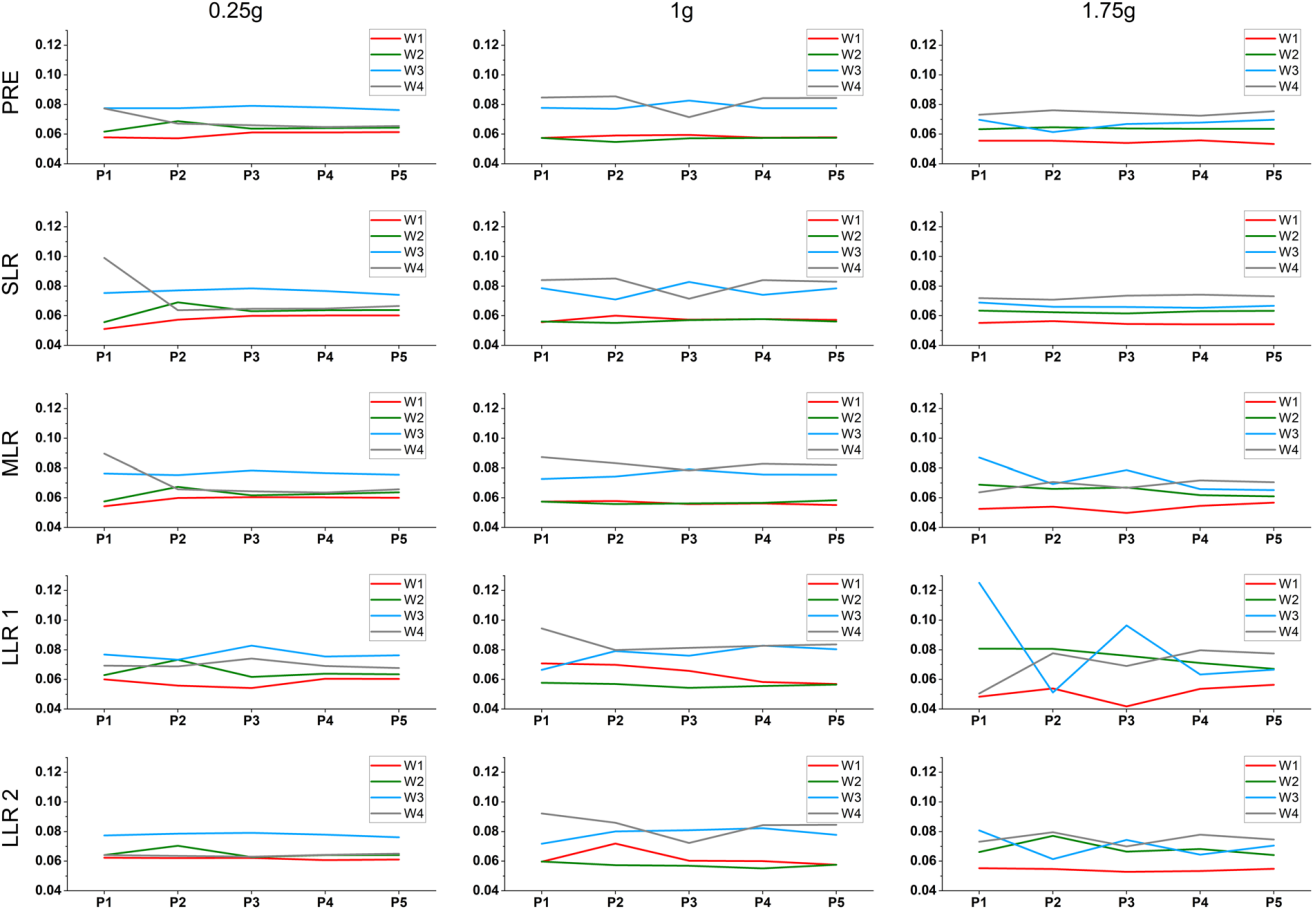
Mean activation coefficients referring to the synergies W1-W4 in Fig. 1 and depending on perturbation type (P1: bilateral posterior perturbation, P2: bilateral anterior perturbation, P3: unilateral posterior perturbation, P4: unilateral anterior perturbation, P5: bilateral perturbation in opposing directions). The tuning curves represent the extent to which each synergy is activated during reflex phases PRE (top), SLR, MLR, LLR1 and LLR2 (bottom) separately.

The extent to which each synergy is activated during perturbations and in different reflex phases is given by the synergy activation coefficients C_i_ and can be seen in Fig. 2: For most gravitational conditions and reflex phases, the synergies W3 and W4 are more activated than W1 and W2 and highest peaks of single synergy activation can be located after posterior perturbations (P1 & P3). With respect to gravitational comparisons, in general the synergy W2 is stronger and the synergy W4 less activated in 0.25 g and 1.75 g than in 1 g. Contrastingly, the synergies W1 and W3 enforce relatively equal activation during SLR and MLR, but their contributions dramatically change during LLR1.

## Discussion

By investigating the synergy organization of postural muscles during balance recovery, this study provides valuable insight into the coordination of motor commands after translational surface perturbation under different gravitational conditions. The study revealed that in 1 g there are 4 principal muscle synergies, each composed by 1 to 4 predominant muscles. The correlation coefficients indicated that there are significant correlations between all corresponding synergies of the 0.25 g and 1 g conditions and between 3 of 4 synergies of the 1.75 g and 1 g conditions. Confirming our hypothesis, the present study therefore emphasizes the similarity of the synergy organization of postural recovery responses in Earth, hypo- and hyper-gravity conditions, indicating that the muscle synergies acquired under terrestrial habits in short-term are robust across different gravity levels.

### Composition of the muscle synergies in earth gravity

Slips and stumbles require a precise neuronal control of postural muscles transmitting the force via the skeleton to the ground in order to produce adequate torques to regain postural equilibrium after deterioration^6,8^. To simplify the coordination of the various antigravity muscles, it has been manifested that the CNS does not control each muscle discretely, but combines certain muscles into synergies, which are inhibited or facilitated in congruence^12–14^. Previous studies using uncontrolled manifold and NNMF approaches showed that multidirectional shifts of the center of pressure can be realized by a small set of only three muscle synergies^13^.

Similar results were found in the present study. In Earth gravity, we found 4 muscle synergies that were each composed by 1 to 4 predominant muscles. Each muscle was active in more than 1 synergy but predominant in not more than 2 synergies. A detailed view on the synergies shows that the synergies W1 and W4 are comprised predominantly of distal leg muscles (GM and TA for W1 and SOL for W4), whereas the W2 and W3 activations are more equally distributed over trunk (RA and ES) and proximal leg muscles (BF for W2 and VM for W3). Within most synergies, agonistic and antagonistic muscles act together stabilizing the body with respect to its environment.

The different postural perturbations in 1 g were mainly counteracted by the synergies W3 and W4, which is indicative of increased trunk and plantarflexor muscle activity when participants are exposed to bilateral and unilateral anterior and posterior surface perturbations^9^. Considering the different reflex phases and directions of perturbations, nonetheless distinct patterns of activation can be identified^19,20^. A detailed analysis suggests a kind of reverse behavior between the trunk (W3) and plantarflexor muscles (W4). While there is a strong activity of the plantarflexors during LLR1 and LLR2 after bilateral posterior perturbations, emphasizing its significance for backward movements of the COM^21^, the trunk muscles seem to compensate for the decreased contribution of W4 during unilateral posterior perturbations. Therefore, one might have argued that the participants have changed their strategy during this kind of perturbation, shifting their weight to the non-displaced right limb in order to have better stability on the leading leg, which would require an increased activity of the trunk and proximal limb muscles (W3). However, as this shift is visible already prior the perturbation (PRE), this explanation is not applicable and it must be assumed that this initial change in synergy contribution persists until MLR. Nonetheless, our results indicate that different synergies might replace each other during balance recovery.

The synergy W2 is rather inactive across all perturbations and reflex phases, while the synergy W1 (plantar- and dorsiflexors) contributes the most during LLR1 and LLR2 after bilateral perturbations. The later finding might be interpreted as a co-contraction strategy, which is employed when both limbs are moved forward or backward.

### Muscle synergies in 0.25 and 1.75 g

The success of regaining postural stability is a result of adequate muscle activation^8,21^, whereas the level of activation depends not only on the size and direction of perturbation, but also on the loading force^6^. Consequently, counteracting an increased gravitational force in the vertical plane concomitant with the compensation of deteriorations in the sagittal plane presupposes an increased neuromuscular activity of postural muscles^6^. According to this, previous studies found that muscle activation intensities as well as timing and magnitude of muscular activation during static posture control undergo remarkable changes from hypo- to hyper-gravity^22–25^. Hence, it is particularly important to investigate muscle activation patterns during reactive balance under different gravitational conditions.

The present results show that the synergies extracted from acute changes in gravity including 0.25 g and 1.75 g are so constituted that every synergy in hypo- and hyper-gravity corresponds with exactly one synergy of the 1 g condition. Correlation coefficients indicated significant interrelations between all corresponding synergies of the 0.25 g and 1 g condition and between 3 corresponding synergies of the 1.75 g and 1 g condition. Even the non-significant pair of synergies (W3) reached a high correlation coefficient.

With regard to the different perturbations and reflex phases, we found quite similar topographies for most synergies and experimental conditions. As under Earth gravitation, perturbations under hypo- and hyper-gravity were mainly counteracted by synergies W3 and W4. However, the plantarflexors (W4) tended to contribute less in 0.25 g and 1.75 g when compared to 1 g. Vice versa, the trunk and proximal limb muscles (W2) were activated stronger in 0.25 g and 1.75 g compared to 1 g in the reflex phases post-perturbation. Hence, the contribution of proximal muscle groups to postural control increases in unfamiliar gravitational conditions, potentially representing a whole-body stiffening that is commonly seen when subjects are exposed to novel balance tasks^26^. The synergies W1 and W3 enforce relatively equal activation during PRE and SLR, but their contributions dramatically change during MLR, LLR1 and LLR2. Specifically in 1 g, synergy W3 appears to be responsible for SLR after posterior perturbations and for LLR1 after anterior displacements; however, in 0.25 g and especially in 1.75 g its contribution during MLR and LLR1 and LLR2 is strongest after posterior perturbations. A similar but reverse behavior can be seen for the plantarflexors (W4), which contribution in 0.25 g is strongest in early reflex phases (SLR, MLR) and after bilateral posterior perturbations, while in 1.75 g its contribution is stronger in later phases with an emphasize on anterior displacements. Hence, there seems to be a gravitational-based adaptation in the different reflexes phase. Particularly, in hypo-gravity there is increased use of the plantarflexors in early reflex phases, whereas in hyper-gravity there is an increased contribution during long latency reflexes, i.e. when sensory information is centrally processed and integrated in postural responses. For W1, adaptations are rather small for early reflex phases, but stronger adaptations are manifested during LLR1, especially in 1.75 g, indicating decreased activity of antagonistic ankle muscles. Nonetheless, no systematic alteration in the synergy organization between the different gravity levels can be identified.

In the context of previous work, which demonstrated a progressive increase in muscle activity and onset latencies with increasing gravity^3^, the current findings emphasize that topographic and strategic factors of motor control remain largely constant across all body segments despite differing loading forces induced by spontaneous changes in gravity in parabolic flights.

## Conclusion

The achievement of a stable posture in various gravity conditions becomes a realistic scenario challenging interplanetary space missions. To acquire empirical knowledge about the effect of altered gravity on static posture control, researchers have investigated the acute effects of micro- and hypo-gravity on the underlying neuromuscular modulation^22–24^. However, to preserve the astronauts’ capability to execute mission-critical tasks and reduce injury risk in transit and on planetary surfaces, a thorough understanding of the motor control of compensatory postural responses after balance deterioration in various gravity conditions becomes indispensable. Even though the use of parabolic flights enabled us to collect only a small set of data providing insight into the acute changes during g transitions, they are the first of its kind and emphasize that the muscle synergies respecting the segmental strategies acquired throughout life time on Earth are valid and robust across variable gravity levels beyond and below 1 g. This finding leads to the assumption that astronaut training during 1 g containing activations of specific muscle groups during recovery of postural deterioration and hence training of the motor control system is transferable into the variable gravitational conditions. Whether there is also a long-term robustness against hypo- and hyper-gravity remains to be investigated. Specifically, future studies must show if there are chronic changes in response to long-term bed rest or space missions.

## Methods and Computational Technique

### Subjects

Five subjects (3 females, 2 males, age 34 ± 8 years, height 173 ± 6 cm, body mass 65 ± 8 kg) volunteered to participate in this study. The participants underwent two medical investigations; exclusion criteria were acute orthopedic injuries or neurological dysfunctions, pregnancy, sickness, vestibular or proprioceptive damage, fear of flying, previous surgeries on the left or right leg and an age >41 years.

All subjects gave written informed consent to the experimental procedure. The study design was approved by the French authorities responsible for the protection of subjects participating in biomedical research (DEMEB of the AFSSAPS) and the ethical committee of the University of Freiburg (89/12). The experiments also complied with current German laws.

### Parabolic flights

**Figure 3:**
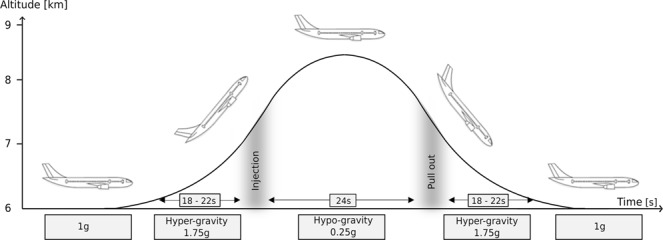
The parabolic flight maneuver and the corresponding gravity levels: the level flight (1 g) becomes a 45° climb flight inducing hyper-gravity (ca. 20 s 1.75 g), followed by hypo-gravity (0.25 g) and another hyper-gravity phase before returning to a level flight. The different gravitational conditions were used to assess neuromuscular control in regular orthograde stance in response to perturbed posture.

Gravitational variation was induced by parabolic flights (PF). The experiment was conducted aboard the ZERO-G aircraft (Novespace, Bordeaux, France) during three PF days. Each PF lasted three hours and comprised 31 parabolas for data recording. However, in contrast to normal PF campaigns, a hypo-gravity level instead of 0 g was provided (Fig. 3).

The recordings during hypo-gravity were conducted during 10 parabolas containing 24 seconds of 0.25 g. The recordings in hyper-gravity (1.75 g) were recorded during the rising and falling periods of the aircraft before and after each parabola, lasting approximately 18–22 seconds each. 1 g measurements were recorded during the outbound or return flight.

Prior to the PF, the participants were injected a sex-weight-based (0.2–0.7 ml) dose of Scopolamine to prevent motion sickness. Based on the data presented by Ritzmann *et al*.^27^, which indicate that the medication with Scopolamine does not impede spinal excitability, maximal motor output and neuromuscular performance parameters (balance and jumps), it can be assumed that the injection also does not affect the muscle synergy organization.

### Perturbations

In each gravity level, six perturbations of five different perturbation modes each were elicited in a random order and with random breaks (3–5 s) according to Dietz *et al*.^28^: (P1) simultaneous bilateral posterior perturbation, (P2) simultaneous bilateral anterior perturbation, (P3) unilateral posterior perturbation of the left leg only, (P4) unilateral anterior perturbation of the left leg only and (P5) simultaneous bilateral perturbation in opposing directions (left leg anterior, right leg posterior). The acceleration impulses were applied independently to the two belts of the treadmill (physical parameters in Table 1) with a software interface coding for randomization (Labview, Imago, Pfitec, Freiburg). The treadmill constituted of two independently moving plates (dimensions: 1.15 m × 0.3 m), each driven by a motor (AKM51G, Danaher Motion, Dusseldorf, Germany). Motor characteristics were as follows: power up to 1200 W; 4.75 Nm torque; 5000 rotations per minute; 400N lateral force. For the purpose of the study and safety regulations of space flight experiments, both plates were secured and foamed. The mechanical displacement was assessed by a potentiometer (sampling frequency 1 kHz). A safety harness secured the subjects from falling.

### Selection of gravity levels

The g-level was monitored by an accelerometer (sampling frequency 1 kHz). The g-data extracted for hypo- and hyper-gravity needed to lay within boundaries of ±0.1 g for a time interval 100 ms prior to until 250 ms after perturbation onset^28^.

### Outcome measures

Bipolar Ag/AgCl surface electrodes (Ambu Blue Sensor P, Ballerup, Denmark, diameter 9 mm, center-to-center distance 34 mm) were placed over the lateral part of the musculus soleus (SOL), gastrocnemius medialis (GM), tibialis anterior (TA), vastus medialis (VM), and biceps femoris (BF) of the left leg as well as over the left musculus rectus abdominis (RA), the lumbar area of the erector spinae left (ES le) and right (ES ri) and the acromial part of the deltoideus (DEL). The longitudinal axes of the electrodes were in line with the presumed direction of the underlying muscle fibers according to SENIAM^29^. The reference electrode was placed on the patella. Interelectrode resistance was kept below 2 kΩ by means of shaving, light abrasion and degreasing of the skin with a disinfectant. The EMG signals were transmitted via shielded cables to the amplifier (band-pass filter 10 Hz to 1 kHz, 200x amplified) and recorded with 1 kHz (A/D-conversion via a National Instruments PCI-6229 DAQ-card, 16 bit resolution). For EMG normalization, subjects performed three isometric maximal voluntary contractions (MVCs) for each recorded muscle according to Roelants *et al*.^30^ and Wiley and Damiano^31^; the trial with the highest EMG activation was used. The MVCs were executed against resistance for 3 s with recovery pauses of 1 min between trials and repetitions. Antagonistic muscle activation was monitored, and trials were repeated when antagonists were activated. Body position during MVCs was strictly controlled and supervised through goniometric recordings with standardized knee and hip joint angles by the authors.

### Data processing

EMG signals were rectified, averaged, integrated and time-normalized for four time intervals, based on previously reported onset latencies and durations of the reflex components^19,20^: the pre-activation phase (PRE, 100–0 ms before perturbation), short latency response (SLR; 30–60 ms after perturbation), medium latency response (MLS; 60–85 ms after perturbation), and long latency responses (LLR1; 85–120 ms after perturbation; LLR2; 120–250 ms after perturbation)^21,32^. These integrals were normalized to the MVC of the corresponding muscle. All data were averaged for identical perturbation modes.

### Extraction of synergies

For analyzing the EMG responses, NNMF was used. MVC normalized EMG data of five subjects were synthesized to one data matrix consisting of 9 rows representing the measured muscles and 125 columns (5 subjects × 5 perturbation types x 5 reflex phases). Matrix forming procedure was done according to Ting and Macpherson^10^ and Chvatal & Ting^9^. The NNMF extracts the muscle synergy vectors W_i_ and synergy activation coefficients c_i_, which represent a particular muscle activity pattern E by

$$E={c}_{1}\ast {W}_{1}+{c}_{2}\ast {W}_{2}+{c}_{3}\ast {W}_{3}+\ldots $$

NNMF uses the algorithm by Lee and Seung^33^, which factors the p-by-n matrix E into non-negative factors W (p-by-s) and C (s-by-n) and minimizes the root mean squared residual D between E and its approximation W * C, starting from random initial values W_0_ and C_0_. This procedure is provided by the MATLAB function nnmf.m^34^ and is given as:

$$D=\frac{{\Vert E-W\ast C\Vert }_{FRO}}{\sqrt{p\ast n}}$$

D represents the p-by-n residual error matrix with p indicating the number of muscles, n the number of time bins and s the number of synergies.$\,\parallel \circ {\parallel }_{FRO}$ yields the Frobenius norm. The number of replicates was set to 10^6^. Iteration was computed with a varying number of synergies from 1 to 9. For the selection of synergies, total variance accounted for was calculated as proposed by Frere and Hug^35^:

$$VA{F}_{tot}=\frac{\sum _{i=1}^{p}\sum _{j=1}^{n}{({e}_{i,j})}^{2}}{\sum _{i=1}^{p}\sum _{j=1}^{n}{({E}_{i,j})}^{2}}$$

As a second criterion for selection of synergies, VAF_cond_ (variance accounted for between two 9-by-5 blocks of the matrices representing the same perturbation type) was computed to ensure that the errors are distributed uniformly over the 5 perturbations^9^. Prevalent lower thresholds for number of synergy selection were 0.9 for VAF_tot_ and 0.75 for VAF_cond_^9,36^.

### Statistics

Similarities between muscle synergies were determined by calculating correlation coefficients (r) between each muscle synergy vector between 0.25 g and 1 g as well as between 0.75 g and 1 g. As r_crit_ = 0.798 indicates the critical value for 9 muscles and p = 0.01, synergies with a higher correlation as r_crit_ were considered as similar^37^. Equivalence statistics were used to determine if the physics of the treadmill pulses were statistically equal between the gravity levels below and above 1 g compared to 1 g. For this purpose, the 95% confidence interval (CI) was calculated for the differences between 1 g and the hypo- and hyper-gravity level. If the CI lay within the acceptable boundaries (which were determined based on the variance within the 1 g data set^38^ the differences were statistically equal and the respective parameter was marked with a ≈ symbol.

## Data Availability

The datasets generated during and/or analyzed during the current study are available from the corresponding author on reasonable request.
